# Marine Products and Their Anti-Inflammatory Potential: Latest Updates

**DOI:** 10.3390/md22080376

**Published:** 2024-08-21

**Authors:** Marzia Vasarri, Donatella Degl’Innocenti

**Affiliations:** Department of Experimental and Clinical Biomedical Sciences, University of Florence, Viale Morgagni 50, 50134 Florence, Italy; marzia.vasarri@unifi.it

The depths of the sea are a rich source of biologically active compounds with therapeutic potential for various human diseases, including inflammatory conditions. The growing need for new and effective health products continuously drives scientific research to discover new marine natural compounds and their biological properties. This fascinating field of study has led to the success of this Special Issue “Marine Anti-inflammatory and Antioxidant Agents 3.0” of the journal *Marine Drugs*, which gathered 13 innovative and original scientific publications. These publications highlight the value of marine natural resources in developing new anti-inflammatory treatments and functional products, aligning with the principles of sustainability and circular bioeconomy, thereby contributing to environmental conservation.

This editorial aims to review recent scientific advances in marine natural products with anti-inflammatory potential, as published in this Special Issue “Marine Anti-inflammatory and Antioxidant Agents 3.0”. It highlights the roles and mechanisms of these products in various applications, including health care for treating inflammation-related disorders, industry for producing functional foods, and environmental conservation within the context of the circular economy. Specifically, this editorial compiles scientific studies on a marine plant, green algae, four brown algae, red algae, two microalgae, and two bacterial strains. Additionally, two reviews are included in this Issue.

Micheli et al. focused their study on the well-known anti-inflammatory properties of the marine plant *Posidonia oceanica* (L.) Delile, demonstrating its potential anti-psoriatic application. Administering an oral hydroalcoholic extract of *P. oceanica* leaves (POE) to C57BL/6 mice with Imiquimod (IMQ)-induced psoriatic dermatitis showed that POE treatment significantly reduced PASI scores, skin thickness, and temperature. Histological improvements and decreased levels of inflammatory cytokines and lipocalin-2 further supported POE’s potential as a natural anti-inflammatory agent for psoriasis, suggesting its possible integration into complementary medicine (Micheli, L.; Vasarri, M.; Deg’Innocenti, D.; Di Cesare Mannelli, L.; Ghelardini, C.; Antiga, E.; Verdelli, A.; Caproni, M.; Barletta, E. *Posidonia oceanica* (L.) Delile Is a Promising Marine Source Able to Alleviate Imiquimod-Induced Psoriatic Skin Inflammation. *Mar. Drugs* **2024**, *22*, 300. https://doi.org/10.3390/md22070300 [1]).

The study conducted by Frusciante et al. represents the forefront in research supporting the circular economy and environmental sustainability. The anti-inflammatory property of an extract obtained from the invasive green macroalga *Chaetomorpha linum*, present in the Orbetello Lagoon, was examined. This alga, typically harvested mechanically and treated as plant waste, revealed unexpected potential. The *C. linum* extract demonstrated potent inhibitory activity on the production of ROS, NO, and PGE2 in cell-based tests, as well as reduced expression of iNOS and COX-2. These results indicate that the extract is promising as a therapeutic candidate for chronic inflammatory conditions like atopic dermatitis. The research underscores the potential value of utilizing underexploited marine biomass for extracting bioactive compounds, offering new opportunities for medical and environmental innovation (Frusciante, L.; Geminiani, M.; Trezza, A.; Olmastroni, T.; Mastroeni, P.; Salvini, L.; Lamponi, S.; Bernini, A.; Grasso, D.; Dreassi, E.; et al. Phytochemical Composition, Anti-Inflammatory Property, and Anti-Atopic Effect of *Chaetomorpha linum* Extract. *Mar. Drugs* **2024**, *22*, 226. https://doi.org/10.3390/md22050226 [1]).

For the first time, Park et al. demonstrated the anti-inflammatory properties of an extract from the brown alga *Sargassum yezoense*, a prevalent species along Korea’s eastern coast, and its fractions. The ethanolic extract significantly reduced inflammation in lipopolysaccharide (LPS)-stimulated RAW 264.7 macrophages and suppressed M1 polarization in bone marrow-derived murine macrophages. Five fractions were obtained through liquid–liquid extraction, with the chloroform fraction (SYCF) showing the highest phenolic content and antioxidant capacity. SYCF, rich in sargahydroquinoic acid (SHQA) and sargachromenol (SCM), exhibited robust anti-inflammatory effects, inhibiting NO production and cytokine expression in macrophages and suppressing NF-κB and MAPK signaling pathways. This indicates SYCF’s potential as a functional food ingredient or therapeutic agent for inflammation-related disorders (Park, Y.; Cao, L.; Baek, S.; Jeong, S.; Yun, H.J.; Kim, M.-B.; Lee, S.G. The Role of Sargahydroquinoic Acid and Sargachromenol in the Anti-Inflammatory Effect of *Sargassum yezoense*. *Mar. Drugs* **2024**, *22*, 107. https://doi.org/10.3390/md22030107 [1]).

Liyanage et al. also used the RAW 264.7 macrophage cell model stimulated with LPS to study the anti-inflammatory properties of fucoidan from the brown alga *Sargassum autumnale*. Among various fucoidan fractions, SAF3 showed the most significant protective effect, inhibiting NO and PGE2 production by downregulating iNOS and COX-2 expression. Additionally, SAF3 reduced the pro-inflammatory cytokines IL-1β, TNF-α, and IL-6, suppressing NF-κB and MAPK signaling pathways in a dose-dependent manner. These results suggest SAF3’s potential as a functional food ingredient or treatment for inflammatory disorders (Liyanage, N.M.; Lee, H.-G.; Nagahawatta, D.P.; Jayawardhana, H.H.A.C.K.; Song, K.-M.; Choi, Y.-S.; Jeon, Y.-J.; Kang, M.-C. Fucoidan from *Sargassum autumnale* Inhibits Potential Inflammatory Responses via NF-κB and MAPK Pathway Suppression in Lipopolysaccharide-Induced RAW 264.7 Macrophages. *Mar. Drugs* **2023**, *21*, 374. https://doi.org/10.3390/md21070374 [1]).

Belonging to the brown algae family, *Ericaria amentacea* was studied by Mirata et al., who analyzed the anti-aging and photoprotective properties of two ethanolic extracts obtained from different parts, namely apices and thalli. Comparing the apices and thalli extracts in HaCaT keratinocyte and L929 fibroblast cell models exposed to UV rays, it was observed that the apices hydroalcoholic extracts showed the highest potential, blocking UV-induced damage and oxidative stress. This study highlighted the importance of *E. amentacea* apices derivatives as ideal components for counteracting sunburn symptoms and for anti-aging cosmetic lotions (Mirata, S.; Asnaghi, V.; Chiantore, M.; Salis, A.; Benvenuti, M.; Damonte, G.; Scarfì, S. Photoprotective and Anti-Aging Properties of the Apical Frond Extracts from the Mediterranean Seaweed *Ericaria amentacea*. *Mar. Drugs* **2023**, *21*, 306. https://doi.org/10.3390/md21050306 [1]).

A formula based on oligo-fucoidan (FF) has shown protective effects against osteoarthritis, reducing inflammation and cartilage damage. The key ingredient, oligo-fucoidan (over 30% of the formula), is derived from the brown algae *Laminaria japonica*. In a study by Chiang and colleagues using a monosodium iodoacetate (MIA) model of osteoarthritis (OA), the formula modulated p38 signaling and reduced COX-2 and iNOS levels. These findings suggest its potential for managing osteoarthritis by improving joint function, reducing inflammation, and protecting cartilage. However, further clinical validation is needed. A better understanding of fucoidan’s therapeutic properties could lead to personalized treatments for osteoarthritis tailored to individual patient needs (Chiang, Y.-F.; Huang, K.-C.; Wang, K.-L.; Huang, Y.-J.; Chen, H.-Y.; Ali, M.; Shieh, T.-M.; Hsia, S.-M. Protective Effects of an Oligo-Fucoidan-Based Formula against Osteoarthritis Development via iNOS and COX-2 Suppression following Monosodium Iodoacetate Injection. *Mar. Drugs* **2024**, *22*, 211. https://doi.org/10.3390/md22050211 [1]).

The edible red alga *Pterocladiella capillacea* is known for its anti-inflammatory properties. Wang et al. demonstrated the xanthine oxidase inhibitory and anti-inflammatory activities of various fractions of *P. capillacea* extract for potential anti-gout applications. These findings support the value of further investigation into *P. capillacea* as part of the development of anti-gout drugs or related functional foods (Wang, Y.; Zhou, L.; Chen, M.; Liu, Y.; Yang, Y.; Lu, T.; Ban, F.; Hu, X.; Qian, Z.; Hong, P.; et al. Mining Xanthine Oxidase Inhibitors from an Edible Seaweed *Pterocladiella capillacea* by Using In Vitro Bioassays, Affinity Ultrafiltration LC-MS/MS, Metabolomics Tools, and In Silico Prediction. *Mar. Drugs* **2023**, *21*, 502. https://doi.org/10.3390/md21100502 [1]).

Research led by Græsholt and colleagues revealed intriguing findings regarding the genetic engineering of the microalga *Phaeodactylum tricornutum* to enhance the production of diatoxanthin, a carotenoid known for its antioxidant and anti-inflammatory benefits. By employing CRISPR/Cas9 gene editing to deactivate the ZEP2 and ZEP3 genes, the study demonstrated that zep3 mutant strains maintained more stable diatoxanthin levels in low-light environments, offering a promising approach for commercial production (Græsholt, C.; Brembu, T.; Volpe, C.; Bartosova, Z.; Serif, M.; Winge, P.; Nymark, M. *Zeaxanthin epoxidase 3* Knockout Mutants of the Model Diatom *Phaeodactylum tricornutum* Enable Commercial Production of the Bioactive Carotenoid Diatoxanthin. *Mar. Drugs* **2024**, *22*, 185. https://doi.org/10.3390/md22040185 [1]).

The marine microalga *Tisochrysis lutea* was also studied for its effects on pre-metabolic syndrome in rats. D’Ambrosio et al. demonstrated that *T. lutea* could reduce triglycerides, glucose levels, and improve adiponectin levels without causing the adverse effects seen with other treatments. The multifaceted impact of *T. lutea* on energy metabolism and inflammation suggests its potential for mitigating metabolic syndrome risks (D’Ambrosio, M.; Bigagli, E.; Cinci, L.; Gencarelli, M.; Chioccioli, S.; Biondi, N.; Rodolfi, L.; Niccolai, A.; Zambelli, F.; Laurino, A.; et al. *Tisochrysis lutea* F&M-M36 Mitigates Risk Factors of Metabolic Syndrome and Promotes Visceral Fat Browning through β3-Adrenergic Receptor/UCP1 Signaling. *Mar. Drugs* **2023**, *21*, 303. https://doi.org/10.3390/md21050303 [1]).

The Gram-positive bacterium *Corynebacterium glutamicum* was the focus of Seeger et al.’s study, who developed an innovative method for ethanol extraction of astaxanthin, a potent antioxidant carotenoid, from genetically modified *C. glutamicum*. Optimal extraction conditions were identified with high recovery and purity of astaxanthin, and the natural astaxanthin showed high antioxidant activity, comparable to or exceeding synthetic variants. This highlights astaxanthin’s potential in cosmetics and nutraceuticals (Seeger, J.; Wendisch, V.F.; Henke, N.A. Extraction and Purification of Highly Active Astaxanthin from *Corynebacterium glutamicum* Fermentation Broth. *Mar. Drugs* **2023**, *21*, 530. https://doi.org/10.3390/md21100530 [1]).

Al-Awadhi et al. identified 7(E)-9-keto-hexadec-7-enoic acid and related analogs from a marine cyanobacterial mat. This compound activated the Keap1/Nrf2 pathway, showing significant anti-inflammatory potential. It reduced nitric oxide levels and modulated inflammatory pathways, suggesting its potential as a dietary intervention for managing inflammation and related diseases (Al-Awadhi, F.H.; Simon, E.F.; Liu, N.; Ratnayake, R.; Paul, V.J.; Luesch, H. Discovery and Anti-Inflammatory Activity of a Cyanobacterial Fatty Acid Targeting the Keap1/Nrf2 Pathway. *Mar. Drugs* **2023**, *21*, 553. https://doi.org/10.3390/md21110553 [1]).

This Special Issue was enhanced by two reviews that offered the latest insights into the anti-inflammatory properties of natural marine products. Khursheed and colleagues present an overview of research on anti-inflammatory compounds derived from marine sources, highlighting their potential as new therapeutic drugs (Khursheed, M.; Ghelani, H.; Jan, R.K.; Adrian, T.E. Anti-Inflammatory Effects of Bioactive Compounds from Seaweeds, Bryozoans, Jellyfish, Shellfish and Peanut Worms. *Mar. Drugs* **2023**, *21*, 524. https://doi.org/10.3390/md21100524 [1]). Meanwhile, Nguyen and co-authors compiled studies on the anti-inflammatory properties of octocorals, summarizing findings from 46 studies conducted between 1995 and April 2023. This review provides a thorough overview of the anti-inflammatory potential of octocorals and aims to inspire further research to develop these compounds into therapeutic agents (Nguyen, N.B.A.; El-Shazly, M.; Chen, P.-J.; Peng, B.-R.; Chen, L.-Y.; Hwang, T.-L.; Lai, K.-H. Unlocking the Potential of Octocoral-Derived Secondary Metabolites against Neutrophilic Inflammatory Response. *Mar. Drugs* **2023**, *21*, 456. https://doi.org/10.3390/md21080456 [1]).

In summary, this Special Issue “Marine Anti-inflammatory and Antioxidant Agents 3.0” emphasized the vast potential of the marine environment as a source of bioactive compounds with promising anti-inflammatory properties. Ranging from plant extracts to carotenoids, fucoidans, and cyanobacterial metabolites, these substances offer new avenues for treating inflammatory diseases and contribute to sustainable therapeutic solutions. The reviewed studies highlight significant advances in understanding the mechanisms of these marine compounds and their potential applications in healthcare, functional foods, and environmental sustainability. Looking forward, continued exploration and research into marine natural products are crucial to fully unlocking their therapeutic potential.
